# Human Adipocyte Conditioned Medium Promotes *In Vitro* Fibroblast Conversion to Myofibroblasts

**DOI:** 10.1038/s41598-020-67175-3

**Published:** 2020-06-24

**Authors:** Mariam Y. El-Hattab, Yoshiaki Nagumo, Francoise A. Gourronc, Aloysius J. Klingelhutz, James A. Ankrum, Edward A. Sander

**Affiliations:** 1 Roy J. Carver Department of Biomedical Engineering, College of Engineering, University of Iowa, Iowa City, IA USA; 2 Department of Plastic Surgery, Kindai University, Faculty of Medicine, Higashiosaka, Osaka Japan; 3 Department of Microbiology and Immunology, Carver College of Medicine, University of Iowa, Iowa City, IA USA; 4 Fraternal Order of Eagles Diabetes Research Center, University of Iowa, Iowa City, IA USA; 5 Department of Orthopedics and Rehabilitation, Carver College of Medicine, University of Iowa, Iowa City, IA USA

**Keywords:** Biomedical engineering, Adult stem cells

## Abstract

Adipocytes and adipose tissue derived cells have been investigated for their potential to contribute to the wound healing process. However, the details of how these cells interact with other essential cell types, such as myofibroblasts/fibroblasts, remain unclear. Using a novel *in-vitro 3D human* adipocyte/pre-adipocyte spheroid model, we investigated whether adipocytes and their precursors (pre-adipocytes) secrete factors that affect human dermal fibroblast behavior. We found that both adipocyte and pre-adipocyte conditioned medium induced the migration of fibroblasts, but only adipocyte conditioned medium induced fibroblast differentiation into a highly contractile, collagen producing myofibroblast phenotype. Furthermore, adipocyte mediated myofibroblast induction occurred through a TGF-β independent mechanism. Our findings contribute to a better understanding on the involvement of adipose tissue in wound healing, and may help to uncover and develop fat-related wound healing treatments.

## Introduction

Impaired wound healing impacts millions of people in the US annually with estimated treatment and management costs totaling $20 billion dollars^1^. Problems in the healing process can arise from multiple factors during any stage of wound healing. Examples include exposure to elevated growth factors and cytokines during a prolonged period of inflammation^2–4^, increased mechanical tension that drives excessive matrix deposition during tissue formation^5–7^, and altered rates of matrix degradation and remodeling during the remodeling phase of wound healing^5,8^. Treatment options include various types of dressings, negative-pressure therapies, laser treatments, and the use of corticosteroids, growth factors, drugs and other cellular products^1,9,10^. The majority of these treatments target keratinocytes, fibroblasts/myofibroblasts, and macrophages^1,2,9,11^, cell types that are each integral to wound healing.

Adipocytes, which are known to secrete free-fatty acids and hormones that regulate metabolism, gluconeogenesis, and inflammation^12,13^, may also be active participants in the healing process^14^. Recent studies have found that adipocytes populate the wound site as soon as 24 hours post-wounding and secrete growth factors and deposit ECM proteins that direct fibroblast activity during wound healing^15,16^. Adipocytes may also be involved in the matrix formation stages of wound healing, as they are closely associated with angiogenesis and production of collagen type VI in tissue from obese patients^17–19^. In addition, a growing number of clinicians have advocated for the use of fat autografts to reduce scarring and improve healing^20–26^. Other adipocyte-related cells, such as their precursors (*i.e*., pre-adipocytes and adipose derived stem cells (ASCs)), may also influence the healing process^20,27–30^.

The details of how adipocytes and adipogenic progenitor cells interact with other essential cell types, such as myofibroblasts/fibroblasts during wound healing, however, remain unclear. The purpose of this study was to explore such interactions by first determining whether secreted factors from human adipocytes and pre-adipocytes cultured in 3D differentially affect dermal fibroblast/myofibroblast behavior in order to further understand the role of adipocyte signaling in wound healing.

## Results

### Production, collection, and administration of PCM and ACM

To determine if adipocytes secrete factors that modulate fibroblast behavior, particularly in the context of wound healing, we collected pre-adipocyte and adipocyte conditioned medium (PCM and ACM, respectively) from an *in vitro* adipocyte spheroid model recently developed by us^31,32^. In this model, mature adipocyte spheroids are generated from immortalized human pre-adipocytes via a scaffold-free method and 10 days of culture in differentiation medium containing IBMX, indomethacin, dexamethasone, and high levels of insulin. These differentiated 3D human adipocyte spheroids were characterized previously^31^, and found to accumulate large lipid droplets with increased differentiation time, secrete adiponectin, and possess high transcript levels for peroxisome proliferator-activated receptor (PPAR-γ), CCAAT/enhancer binding protein-α (CEBPα), fatty-acid binding protein 4 (FABP-4), and adiponectin (all markers of adipocyte differentiation^12,33–35^) after 10-days of differentiation.

After 10 days, adipocyte and pre-adipocyte spheroids were removed from either differentiation media or pre-adipocyte growth media, respectively. Spheroids were then washed with PBS and cultured in DMEM containing 0.5% fetal bovine serum (FBS) for 2 days to allow for the collection of secreted factors. This conditioned media (*i.e*., ACM and PCM) was collected and used undiluted for subsequent experiments.

### PCM and ACM increased scratch closure

**Figure 1:**
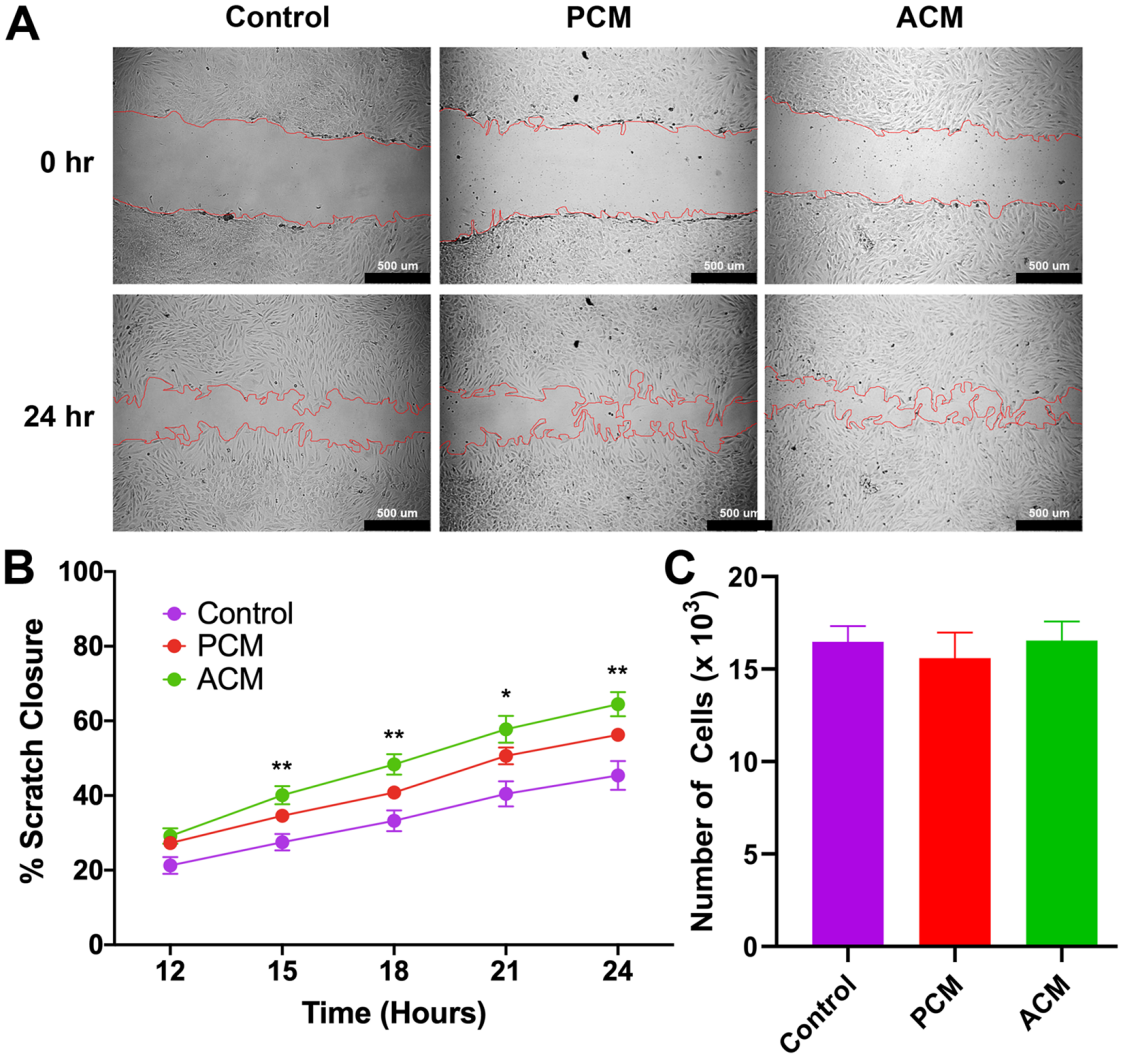
Adipocyte Conditioned Medium Promotes Fibroblast Scratch Closure. (**A**) Representative 10x DIC images at 0 and 24 hours of human dermal fibroblasts in control medium, pre-adipocyte conditioned medium (PCM) and adipocyte conditioned medium (ACM), respectively. Visually identified scratch boundaries are demarcated in red. Scale bar is 500 µm. (**B**) Quantification of percent scratch closure. Data is presented as mean $\pm$ SEM (n = 6 independent experiments with four samples per group). A two-way ANOVA with Dunnett multiple comparison tests at each time point indicate that fibroblasts exposed to ACM closed the gap significantly faster than fibroblasts in control medium (**p<0.01, *p<0.05). (**C)** Cell number after 48 hours was not significantly different between groups, indicating that ACM does not enhance proliferation but promotes fibroblasts closure of the scratch.

In order to determine if ACM and PCM contain factors that modulate fibroblast migration, we first performed a simple scratch assay on confluent monolayers of fibroblasts (Fig. 1A). After 24 hours, ACM treated samples closed the gap (64.5% ± 3.2%, mean ± SEM) to a significantly greater extent (p < 0.01) than control (45.4% ± 3.8%) but not compared to PCM treated samples (56.3% ± 1.9%).

In addition to the final scratch closure being similar between the ACM and PCM groups, the rate of closure was also very similar. The rate of scratch closure (Fig. 1B) over a 24-hour period was relatively constant (i.e., linear) with rates determined by linear regression of 0.310 mm^2^/day, 0.297 mm^2^/day, and 0.229 mm^2^/day for ACM, PCM, and control, respectively. As scratches can close due to both cell migration as well as cell proliferation, we measured the rate of proliferation of fibroblasts exposed to ACM, PCM, or control media for 48 hours. We found no difference in the proliferation rate between the 3 conditions (Fig. 1C), suggesting the difference in closure is not due to enhanced proliferation from secreted factors in conditioned media.

### ACM increased fibrin gel compaction and fibroblast contractility

**Figure 2:**
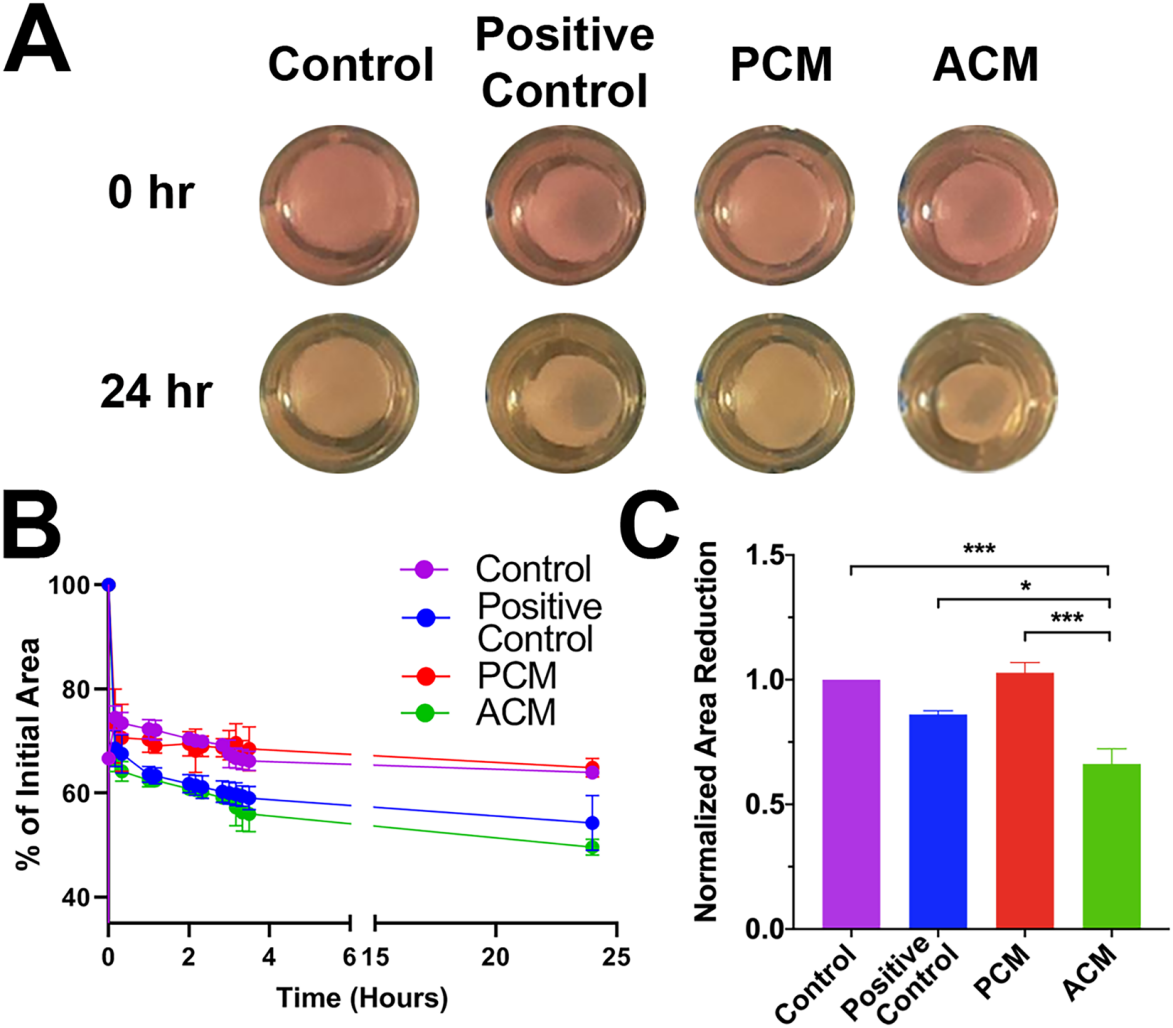
Adipocyte Conditioned Medium Promotes Fibrin Gel Compaction. (**A**) Representative images of fibroblast-seeded fibrin gels shortly after release and 24 hours later. Gels were cultured in control media, TGF-β1 and AA supplemented media (positive control), pre-adipocyte conditioned media (PCM), or adipocyte conditioned media (ACM). (**B**) A representative experiment showing percent reduction in initial gel area (*i.e*., compaction) over time (mean $\pm$ SD, n = 3). (**C**) Fibrin gel compaction at 24 hours normalized by controls for n = 3 independent experiments, each with 3 sample replicates per group. Data is presented as mean $\pm$ SEM. A one-way ANOVA with Tukey post hoc tests indicates ACM treated gels were significantly reduced in area compared to control (p < 0.001), positive control (p < 0.05), and PCM (p < 0.001).

We next asked whether ACM contains factors that modulate fibroblast to myofibroblast conversion. Fibroblasts convert to myofibroblasts most commonly in response to biochemical and mechanical cues in the wound, such as transforming growth factor-β1 (TGF-β1) and mechanical tension^36,37^. Myofibroblasts are a highly contractile and synthetic phenotype characterized by an abundance of cytoskeletal α-smooth muscle actin (α-SMA)^8,37–39^. To test first for an increase in functional contractility, we performed a gel compaction assay (Fig. 2A) where we exposed fibroblast-populated fibrin gels to ACM or PCM for 48 hours, released the gel from the edges of the well, and measured the change in gel area^40,41^. All samples rapidly decreased in area post-release, with compaction in control and PCM samples proceeding for approximately seven hours before plateauing. Positive control (TGF-β1 and ascorbic acid) and ACM treated samples both compacted at a faster rate and to a greater extent than control and PCM samples (Fig. 2B). ACM gel area at 24 hours (normalized by controls) decreased significantly more than the PCM (p < 0.001), control (DMEM with 0.5% FBS) (p < 0.001), and positive control gels (p < 0.05) (Fig. 2C). We were particularly surprised to see that ACM led to higher compaction compared to the positive control, which contained 1 ng/mL TGF-β1 and 50 µM ascorbic acid (AA), concentrations known to increase gel compaction, collagen production, and myofibroblast conversion^41–44^.

### ACM increased cytoskeletal α-SMA expression in fibroblasts

**Figure 3:**
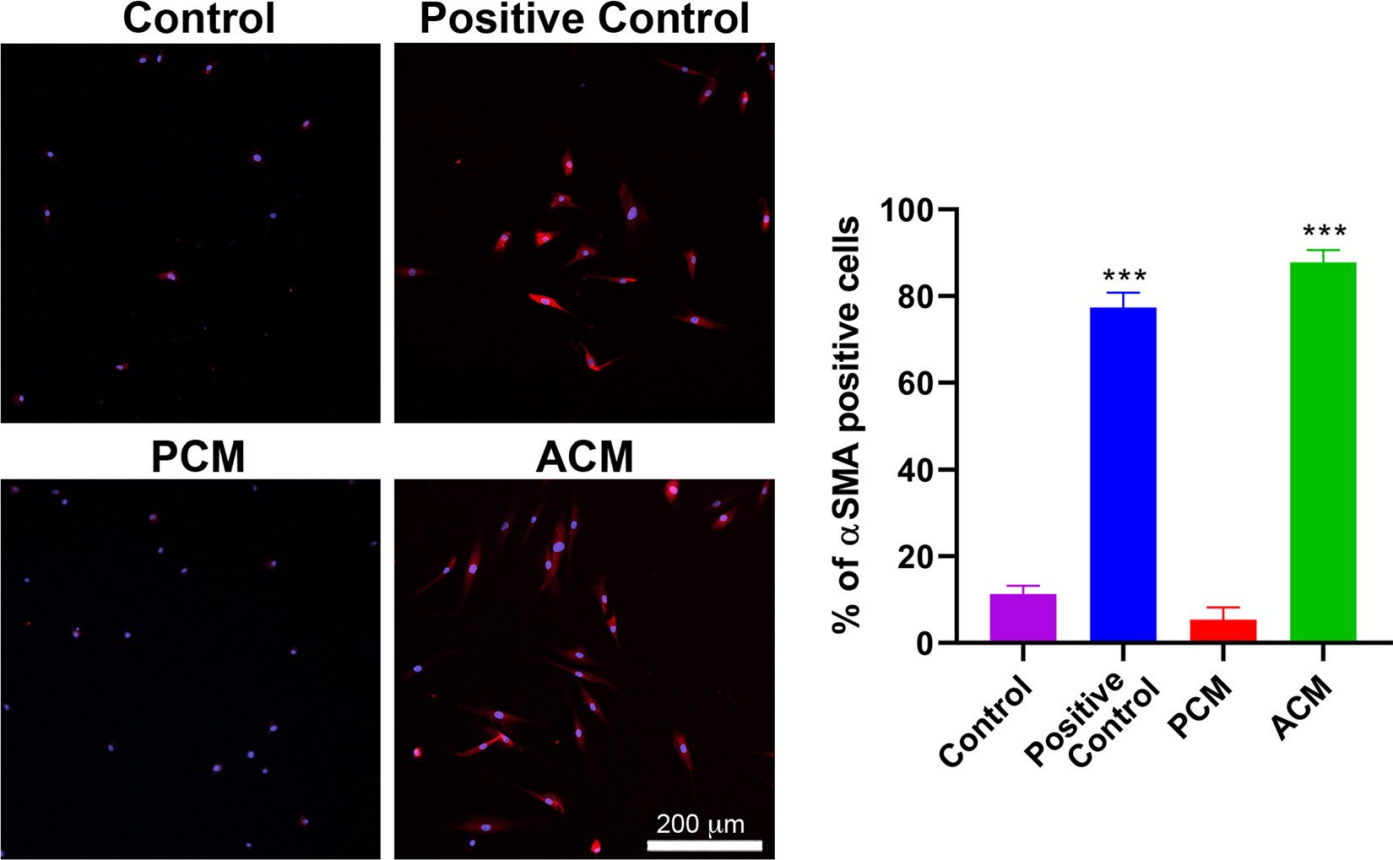
Adipocyte Conditioned Medium Converts Fibroblasts to Myofibroblasts. Representative images showing the extent of α-SMA labeling (red) after 48 hours of exposure to control medium (control), TGF-β1 and AA (positive control), pre-adipocyte conditioned media (PCM), or adipocyte conditioned media (PCM). Cell nuclei labeled with are shown in blue. One-way ANOVA with Tukey post hoc tests (***p < 0.001) indicate that the percentage of α-SMA positive cells was significantly higher for positive control and ACM compared to control and PCM (mean $\pm \,$SD, n = 3).

To check if the increase in compaction was associated with an increase in α-SMA expression, we immunolabeled the gels from each of the four culture conditions and only found an abundance of α-SMA positive fibroblasts in positive control and ACM gels. To facilitate imaging and quantification, we also cultured fibroblasts in 2D on glass chamber slides with the same four culture conditions used in the compaction assay for 48 hours. We then measured the fraction of α-SMA positive cells (Fig. 3) and found that a significantly (p < 0.001) larger percentage (85.8% ± 7.8%) of the fibroblasts cultured with ACM were α-SMA positive when compared to PCM and controls (8.9% ± 1.9% and 7.5% ± 0.8%, respectively). The percentage of α-SMA positive cells cultured with ACM was similar to positive controls (79.0% ± 3.1%).

### ACM increased fibroblast collagen synthesis in fibrin gels

**Figure 4:**
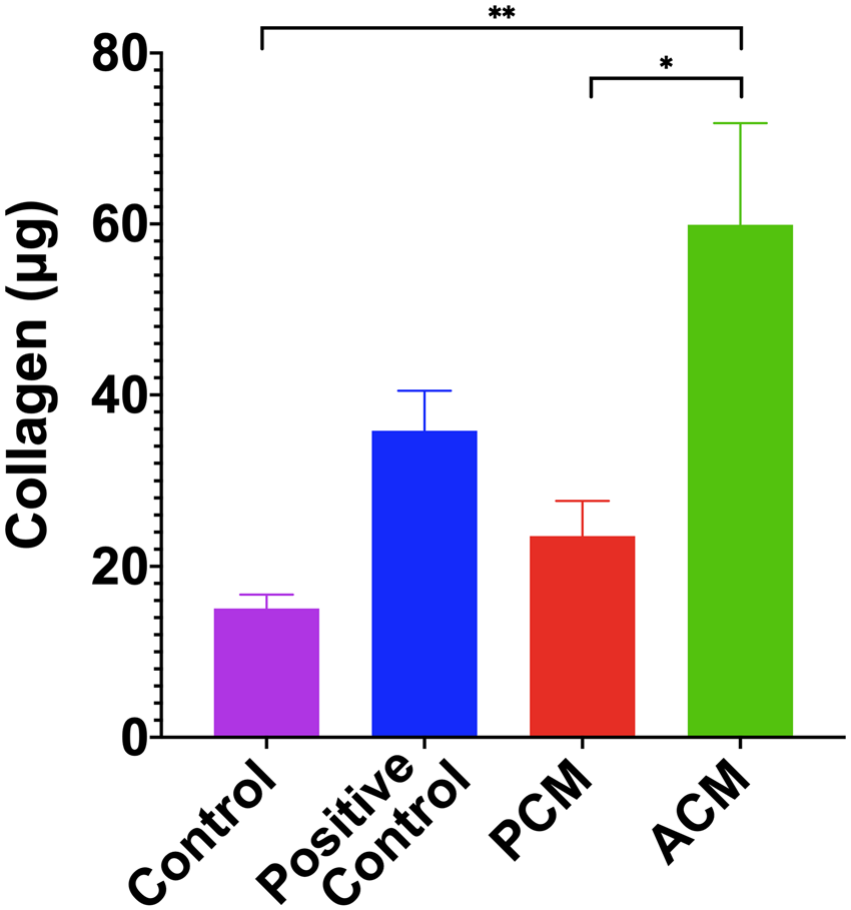
Adipocyte Conditioned Medium Stimulates Fibroblast Collagen Synthesis. ACM treated fibroblasts produced more than three times as much total collagen as controls and 1.5 times as much collagen as positive controls treated with TGF-β1 and ascorbic acid. A one-way ANOVA with Tukey post hoc tests indicates significant differences (*p < 0.05, **p < 0.01). Data is presented as mean ± SEM (n = 3 independent experiments with three replicates for each group).

Because increased collagen synthesis is another hallmark of myofibroblasts in a healing wound^45–47^, we also measured collagen content in fibroblast-populated fibrin gels at the end of nine days of culture. The total amount of collagen per gel (mean ± SEM) for control, positive control, PCM and ACM media was 15.1 ± 1.6 μg, 35.8 ± 4.7 μg, 23.5 ± 4.1 μg, and 59.9 ± 11.9 μg, respectively (Fig. 4). ACM treated samples consistently produced 1.5 to four times as much collagen than all other medium conditions, with significantly higher production than control (p < 0.01) and PCM (p < 0.05).

### TGF-β1 in ACM was not responsible for conversion to a myofibroblast phenotype

**Figure 5:**
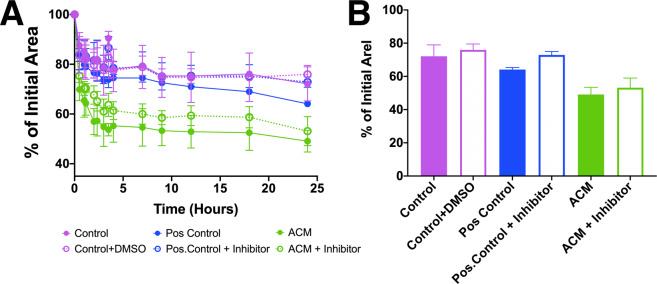
Inhibition of TGF-β1 receptor using SB505124 reduces but does not diminish fibroblast mediated compaction of fibrin gels treated with ACM. (**A**) Percent compaction of fibrin gels after 24 hours treated with control, control with DMSO, positive control, positive control with inhibitor, ACM, and ACM with inhibitor (mean $\pm$ SD, n = 3). (**B**) Compaction of the gels at 24 hours. ACM treated and inhibited samples compacted the most after 24 hours (46.21% and 56.24%, respectively), with ACM inhibited samples still compacting more than positive control and positive control with inhibitor (68.8% and 73.2%, respectively).

These findings indicate that ACM but not PCM contains factors that promote fibroblast to myofibroblast conversion. We next wanted to determine if TGF-β1 in the ACM could be responsible for the myofibroblast phenotype. Because there are reports that adipocytes secrete TGF-β1^18,48,49^, we repeated the gel compaction assay with the addition of 1 µM SB505124, a competitive inhibitor that binds to the TGF-β1 R1 receptor^50^. While SB505124 reduced gel compaction when used with the positive control, to our surprise we found that fibroblast-populated gels treated with ACM and inhibitor compacted similarly to their counterparts (53.1% ± 5.8% and 49.1% ± 4.3%, respectively), suggesting that TGF-β1 is not primarily responsible for the effects reported here (Fig. 5). We also measured TGF-β1 in the medium with an ELISA. We found the levels of TGF-β1 to be similar in the PCM (104.0 ± 14.1 pg/mL) and ACM (139.9 ± 10.0 pg/mL), with each roughly 10% of the 1 ng/mL added to positive controls, and not significantly different from each other. These levels were, however, significantly (p < 0.01) higher than the 11.4 ± 1.3 pg/mL detected in DMEM with 0.5% FBS, which indicates that: (1) both pre-adipocytes and adipocytes secrete low amounts of TGF-β1 into the medium, and (2) that there are other unidentified factor(s) in ACM that are responsible for converting fibroblasts into myofibroblasts.

## Discussion

A growing body of evidence, both in terms of basic research and clinical practice, indicates that adipose tissues contribute to tissue repair and restoration. Lineage tracing indicates that adipocytes repopulate the wound site during the middle to late stages of wound healing (*i.e*., tissue formation and maturation) when fibroblasts are most active^16^. When adipogenesis is inhibited, fibroblast density and activity diminish substantially, suggesting that there are strong interactions between adipocytes and other cell phenotypes, such as fibroblasts, in a healing wound^16^. In fact, several recent reports also utilizing lineage tracing and incorporating flow cytometry and single-cell RNA sequencing have found a number of complex direct and indirect interactions between adipocytes and fibroblasts during wound healing^51–54^.

Pre-adipocytes also secrete factors that can promote healing^27,55^. For example, pre-adipocytes in a cutaneous reconstruction model promoted keratinocyte growth, organization, and differentiation^27^. Additionally, pre-adipocyte factor-1 has been shown to have elevated expression in wounds attaining full regeneration^55^. ASCs have been found to contribute to angiogenesis, prevent fibrosis and early apoptosis^20^, and secrete factors that promote fibroblast proliferation and migration^28–30^. Alternatively, local injection of adipokines or adipocyte secreted factors also appear to improve the healing response^15^. Such findings suggest that adipocyte-related proteins, cells, and tissues play a central role in normal wound healing.

Clinically, autologous fat grafting has been adopted widely in reconstructive surgery because of its utility in tissue reconstruction and augmentation^25,26,56^. More recently, lipoaspirate, processed to retain different components of the stromal vascular fraction (SVF), has been promoted for its potential to improve healing and reverse scarring from traumatic injury^23^, burns^21,22^, and radiation^25,26^. There is, however, uncertainty regarding the efficacy of this procedure, and the underlying biological processes involved remain largely unknown. For example, it is not clear whether adipocytes, pre-adipocytes, ASC, or other factors contained within the fat graft are the primary driver of the reported improvements in tissue remodeling^23,57^.

Furthermore, the fat grafting field is fragmented in the methods applied and terminology used to describe the cells under investigation. Studies that describe the same techniques for cell isolation also describe the resultant cells using terms like SVF, ASC, and adipose-derived mesenchymal stem cells (adMSC), often with little or no characterization of the cells being used. In reality, freshly isolated adipose stromal vascular fractions contain a variety of adipose precursor cells, fibroblasts, endothelial cells, and immune cells. In addition, if SVF is cultured, the prevalence of each cell type in the resultant outgrowth is heavily dependent on the culturing environment and media supplements utilized to support cell expansion. As each of these cell types may have a unique contribution to wound healing environments, it is important to uncover how different subsets of the SVF impact the behavior of fibroblasts within the wound environment.

In this study, we examined interactions between fibroblasts and secreted factors from two components of the SVF: pre-adipocytes and adipocytes that have just matured but not yet accumulated large lipid droplets that would exclude them from the SVF. We found that adipocyte spheroids, but not pre-adipocyte spheroids, secrete factors that increased fibroblast scratch closure, contractility, α-SMA expression, and collagen production, all of which indicates ACM contains factors that convert fibroblasts to myofibroblasts.

Normal tissue repair requires the recruitment of fibroblasts to the site of injury. These recruited fibroblasts, which are attracted by a range of stimuli and can originate from multiple cell sources^38^, transition into highly contractile and synthetic cells that remodel and repair the surrounding tissue. Myofibroblasts are thought to programmatically disappear via apoptosis once tissue integrity is restored^37,58–60^. The most common pathway for myofibroblast conversion is through an increase in elevated mechanical tension^5–8^ coupled with activation via TGF-β1^61–63^. Therefore, we hypothesized that adipocyte-secreted TGF-β1 might be responsible for the observed effects on fibroblasts. Instead, we found that adipocytes and pre-adipocytes secreted similar (but relatively low) amounts of TGF-β1, consistent with Choy *et al*^49^. Furthermore, a TGF-β1 inhibitor did not abrogate the effects of ACM, which indicates that other unidentified adipocyte-secreted factors must be involved. Other mechanisms of fibroblast to myofibroblast conversion are possible, either acting alone or in conjunction with TGF-β1, including other cytokines (e.g., Wnt, angiogenstin II, IL-13, IL-4, CTGF/CCN2, endothelin-1^64–68^), reactive oxygen species^68,69^, microRNAs (e.g., miR-21 and miR-145)^70–72^, and mechanical environment (e.g., matrix stiffness/mechanical tension)^73–75^.

The study of adipocyte-fibroblast interactions is a rapidly emerging area of research and some reports have shown that adipose tissue-derived cells secrete factors that alter fibroblast activity. Ezure and Amano found that enlarged adipocytes (but not small adipocytes) in 2D *in vitro* culture decreased fibroblast collagen (*COL1A1)* and elastin (*ELN)* gene expression and increased matrix metalloproteinase (*MMP13)* gene expression via palmitic acid secretion^76^. This free fatty acid activated toll-like receptors and induced NF- κB translocation to the nucleus, a process that suppresses myofibroblast conversion^77^. These results suggest that adipocytes secrete factors that reduce fibroblast matrix production, a finding opposite of ours. Major experimental differences between studies could explain this discrepancy, such as differences in: (1) adipocyte secretion profiles in 2D adherent versus 3D spheroid culture; (2) adipocyte source (mouse 3T3-L1 cells versus human pre-adipocytes), (3) bidirectional cell-cell communication in co-culture versus unidirectional cell communication with conditioned medium, and (4) adipocyte maturity. With respect to maturity, enlarged adipocytes represented adipocytes 21 days after induction. Our adipocytes, which have matured but not accumulated large lipid droplets, were used 10 days after the initiation of differentiation, a time frame closer to that of Ezure and Amano’s small adipocytes (8 days of induction). Interestingly, these small adipocytes secreted more adiponectin than enlarged adipocytes^78^. In addition, Ezure and Amano, in an earlier study, found that exogenous adiponectin increased dermal fibroblast production of type I collagen in a concentration-dependent manner, but without a change in *COLA1A* gene expression^79^. Our adipocyte spheroids (but not pre-adipocyte spheroids) secrete adiponectin^31^, which suggests that adiponectin could be one of the components of ACM that is responsible for the effects observed in our study, which warrants further investigation.

Although our data reveals an interesting interaction between adipocytes and fibroblasts that does not depend on TGF-β1, additional work is needed to identify the factor or factors responsible and to uncover what role these interactions play in wound healing. Ongoing areas of investigation include fractionating ACM to screen for the biomolecule(s) of interest via properties, such as size, solubility, stability, *etc*., prior to conducting a more detailed -omics analysis, and assessing whether the application of ACM in a pig burn model offers therapeutic benefits that improve wound healing. With respect to the latter, it will be interesting to see if ACM modulates myofibroblast activity in a way that reduces scarring, akin to the purported improvements associated with autologous fat grafting. Paradoxically, elevated myofibroblasts activity is usually associated with increased wound contraction and scarring, and many strategies have focused on limiting myofibroblast activity^73,80,81^.

However, recent work suggests myofibroblasts are far more complex and nuanced in their phenotype and function than previously thought. Distinct myofibroblast subpopulations have been found in healing wounds^52–54^ and some evidence suggests that these subpopulations have unique functions in tissue repair. For example, Shook *et al*. found that myofibroblasts originating from neighboring dermal adipocytes were enriched with many wound healing associated genes including ECM molecules shared by other myofibroblast subtypes, but not for genes associated with collagen maturation and cross-linking^52^ Such results offer the possibility that scarring is more a function of which myofibroblast populations are most active during the healing process.

In conclusion, we found that 3D adipocyte spheroids secrete factors that convert dermal fibroblast to myofibroblasts in a TGF-β1-independent manner. Future work will identify what these factor(s) are and the role they play in wound healing and scarring. Such information will contribute to the growing body of knowledge regarding adipocytes and be useful for improving the use of adipose tissue products applied to various wound healing applications.

## Methods

### Human cells

Primary human dermal fibroblasts (HDF) from 23-year-old and 28-year-old donor were obtained from LifeLine Technologies (FC-0024 Lot: 03869) or breast surgical discard from the University of Iowa Hospitals and Clinics, respectively. To isolate skin fibroblasts, skin pieces were disinfected and treated with dispase overnight and the epidermis was removed. The dermis was then digested with type II collagenase to completion followed by centrifuging and several washing steps before plating in 10% FBS/DMEM. Primary fibroblasts were utilized between passages 3 and 9. Immortal human fibroblasts were made by transducing cells with a TERT-expressing retrovirus as previously described^82^. For imaging in scratch wound assays, the cells were made to express cherry fluorescent protein by transducing with a lentiviral vector that expresses mCherry followed by cell sorting. Immortalized human pre-adipocytes have been previously described^31,32,83,84^. The pre-adipocytes were derived from a non-diabetic female donor. Tissue for isolation of cells was obtained through the University of Iowa Tissue Procurement Core Facility under a University of Iowa Institutional Review Board approved protocol (IRBs 201103721 and 199910006) in accordance with the Department of Health and Human Services regulations 45 CFR 46. Written informed consent was obtained from each donor. Tissue samples were deidentified before a transfer to our lab, and all subsequent experiments using the isolated cells were performed in accordance with the usage agreement.

### Pre-adipocyte & adipocyte spheroid preparation and conditioned medium collection

Pre-adipocyte spheroids were generated using a standard hanging drop cell culture method as described previously, and maintained in pre-adipocyte growth medium (PGM2) containing pre-adipocyte basal medium (Lonza PT-8002), 10% fetal bovine serum (FBS), 0.01% gentamycin sulfate /amphotericin B, and 200 µM L-glutamine^31^. Pre-adipocyte spheroids, each containing ~ 20,000 cells, were then maintained (*i.e*., the maintenance phase) for 10 days in ultra-low adherent 24-well plates (Corning CLS3473), with five spheroids in each well. In parallel, adipocyte spheroids were generated by growing pre-adipocyte spheroids in pre-adipocyte differentiation medium (PDM2) containing 1% insulin, 0.1% 3-isobutyl-1-methylxanthine (IBMX), 0.1% dexamethasone, and 0.2% indomethacin for 10 days (*i.e*., differentiation phase)^31^. After 10 days, the maintenance or differentiation medium (for pre-adipocyte and adipocyte spheroids, respectively) was discarded. The spheroids were rinsed gently with 1X PBS. Next, 0.75 mL of Dulbecco’s Modified Eagle Medium (DMEM) containing 0.5% fetal bovine serum (FBS, Gibco 26140079), 1% penicillin/streptomycin (PS, Gibco 15140-122) and 0.1% amphotericin B (AB, Gibco 15290-018) was added to each well. After two days, this medium, termed either pre-adipocyte conditioned medium (PCM) or adipocyte conditioned medium (ACM) was collected and stored at −80 °C.

### Cell proliferation assay

To quantify cell proliferation in low serum medium and the four treatment groups, cells were plated in a 24-well plate 7,000 cells/cm^2^. They were left to grow for two days in each treatment and trypsinized using 0.25% trypsin-EDTA. The cell solutions were centrifuged at 300 xg for 5 minutes and resuspended in 1x PBS with Hoechst dye (Life Technologies 33342) at a concentration of 5 mg/mL. The cell solutions were transferred to a 96-well plate and fluorescence was measured at 390 nm on a Thermo Scientific Varioskan LUX plate reader.

### Scratch assay

Fibroblasts were cultured in 48-well plates at a density of approximately 20,000 cells/well and in DMEM with 10% FBS 1% PS, and 0.1% AB. After 24 hours when cell confluency reached 80-90%, medium was replaced with either DMEM containing 0.5% FBS and 1% PS and 0.1% AB (control), PCM, or ACM. A sterile 100 µl pipette tip was used to gently scratch a 100 μm wide cell-free gap in the cell monolayer. Preliminary experiments revealed the rate of scratch closure was highest after the initial 12 hours, so the scratch was imaged every 3 hours from t = 12 hours to t = 24 hours with a Nikon inverted DIC microscope at 10x magnification. Scratch closure was quantified by measuring the cell-free area in ImageJ (National Institutes of Health, Bethesda, MA) and calculating an area reduction ratio at each time point.

### Fibrin gel compaction assay

Fibroblasts were suspended homogeneously at a concentration of 500,000 cells/mL in 6 mg/mL fibrin gels. 0.5 mL gels were polymerized in a 24-well plate. Each gel was supplied with 0.5 mL of either (1) DMEM containing 0.5% FBS, 1% PS and 0.1% AB (control), (2) positive control medium that also included 1 ng/mL TGF-β1 (100-21 C, Peprotech, Rocky Hill, NJ) and 50 μg/mL ascorbic acid (Fischer Chemical, A61-25) to induce myofibroblast conversion, (3) PCM, or (4) ACM. After 48 hours, the gels were released gently from the edges of the wells using a sterile spatula and the reduction in gel area (*i.e*., compaction) was observed and measured for an additional 24 hours. Gel area changes were quantified using the free-hand area tool in ImageJ. The temporal change in gel area was normalized to the area change in acellular gels (to account for any reduction in area from the spatula). Data are presented as mean $\pm$ standard deviation (n = 9 per group).

### Identifying myofibroblast presence via α-smooth muscle actin immunolabeling

Fibroblasts were seeded at 20,000 cells/cm^2^ in 4-well glass bottom chamber slides (Lab-Tek 177399) in DMEM containing 10% FBS, 1% PS, and 0.1% AB. Cells were allowed to attach for 6–8 hours and then the high serum medium was removed. Cells were then rinsed once with PBS, and replaced with the four previously defined treatment groups. Samples were cultured for 48 hours at 37 °C and 5% CO_2_ in their respective treatment groups. Samples were then rinsed with PBS, fixed with 4% paraformaldehyde and permeabilized with 1% TritonX-100. Samples were blocked in 5% BSA-tween solution and incubated with a primary mouse anti-human α-SMA antibody (ab7817, Abcam, Cambridge, UK) solution at a 1:100 dilution overnight at 4 °C. Samples were blocked again and incubated with an anti-mouse IgG secondary antibody conjugated to AlexaFluor 568 before a final rinse with PBS. Vectashield mounting medium containing DAPI stain (Vector Labs H-1200) was added to the samples prior to imaging. Samples were imaged using an inverted Nikon Ti2 Eclipse A1 confocal microscope. The percentage of α-SMA positive cells was quantified using CellProfiler software (BROAD Institute). For each media condition, three representative images were quantified, and the experiment was repeated twice with primary fibroblasts from different donors.

### Collagen quantification

Fibroblast seeded fibrin gels were prepared in 24-well plates and cultured with either (1) control medium, (2) positive control medium, (3) PCM, or (4) ACM for 9 days in order to quantify collagen production^41,85^. During this period, the medium was replaced every 3 days. Gels were then prepared for collagen quantification using the hydroxyproline assay, as described previously^85^. Briefly, gels were lysed with 1 N NaOH at 98 °C for 1 hour before lyophilizing in a rotary evaporator for 1 hour. 6 N HCl was then added to the samples and heated to 110 ^o^C for 24 hours and lyophilized as before. Samples were resuspended in assay buffer and filtered through activated charcoal. Samples were then centrifuged at 16,000 x g for 25 minutes to pellet the charcoal. The two-step colorimetric assay then began with the addition of pre-prepared Chloramine-T solution and an incubation of 15 minutes at room temperature, followed by addition of para-dimethylaminobenzaldehyde (pDMBA) solution with a 30-minute incubation at 37^°^C. Absorbance was measured at 570 nm on a Thermo Scientific Varioskan LUX plate reader. Collagen content was then estimated by assuming 7.46 µg collagen/µg hydroxyproline measured^85^.

### TGFβR1 Antagonist experiments

To determine whether the effects of ACM medium on fibroblasts are attributable to adipocyte secreted TGF-β1, the compaction assay was repeated with 23-year-old HDFs in the presence of SB505124 (#3263, TOCRIS), a small molecule that inhibits the TGF-β type I receptor. For these experiments, gels were cultured with the four treatment groups as before, but with the addition of 1μM SB505124^50^.

### TGF-β1 ELISA

To measure TGF-B1 concentration, a sandwich ELISA (#88-8350-22, ThermoFisher) was performed in triplicate on ACM, PCM, control (DMEM with 0.5% FBS), and DMEM following the protocol supplied by the manufacturer.

### Statistics

All statistical analysis was conducted in GraphPad Prism version 8.1.2 (GraphPad, San Diego, CA). Replicates from independent experiments were used to determine individual experiment means, which were then analyzed for significance (p < 0.05) using either one-way or two-way analysis of variance (ANOVA) followed by either Dunnett’s multicomparison tests or Tukey *post hoc* tests.

## Data Availability

The datasets generated during and/or analyzed during the current study are available from the corresponding authors on reasonable request.
